# Editorial Comment: Does YouTube include high-quality resources for training on laparoscopic and robotic radical prostatectomy?

**DOI:** 10.1590/S1677-5538.IBJU.2020.02.08

**Published:** 2020-01-10

**Authors:** Eliney F. Faria

**Affiliations:** 1 Serviço de Urologia Hospital de Amor BarretosSP Brasil Serviço de Urologia, Hospital de Amor de Barretos, SP, Brasil;; 2 Serviço de Urologia Hospital Felicio Rocho Belo HorizonteMG Brasil Serviço de Urologia, Hospital Felicio Rocho – Belo Horizonte, MG, Brasil

Arslan B ^1^, Gönültaş S ^2^, Gökmen E ^2^, Özman O ^2^, Onuk Ö ^3^, Yazıcı G ^2^, Göv T ^2^, Özdemir E ^2^

^1^ Department of Urology, Istanbul Gaziosmanpasa Taksim Training and Research Hospital, Karayolları Str. No: 621 Gaziosmanpaşa, Istanbul, Turkey; ^2^ Department of Urology, Istanbul Gaziosmanpasa Taksim Training and Research Hospital, Karayolları Str. No: 621 Gaziosmanpaşa, Istanbul, Turkey; ^3^ Department of Urology, Yeni Yüzyıl University, Istanbul, Turkey

World J Urol. 2019; 9. [Epub ahead of print]

**DOI:** 10.1007/s00345-019-02904-6 | **ACCESS:** 10.1007/s00345-019-02904-6

## COMMENT

In this interesting paper Dr. Arslan from Turkey showed the increasing use of online resources to seek visual information for both patients and health care providers. The YouTube has become the second largest search engine with more than one billion users and six billion hours of video watched for each month (1). The authors assessed the quality of the YouTube video content related to conventional laparoscopic radical prostatectomy (LRP) and Robotic-assisted radical prostatectomy (RARP). Of the 1,688 videos collected from YouTube content, 226 videos were analyzed (were excluded duration less than 3 min, duplicate and/or irrelevant videos). They were evaluated regarding a scoring tool, named as Prostatectomy Assessment and Competency Evaluation (PACE) score, which was developed and validated by Hussein et al. (2). They evaluated critical steps using a 5-point scale. The median of video length and median of views were 10 minutes and 586 views for LRP, and 22 minutes and 742 views for RARP. The majority of the videos (70.3%) were uploaded by a medical professional. The open access nature of YouTube allows great interaction for upload and views. They showed that longer video might predict the high-quality scores because there is more time to present all steps of the operation systematically. They concluded YouTube website includes high-quality videos for both laparoscopic and robot-assisted radical prostatectomy, and can help in future the development of training programs.
